# MLIF Modulates Microglia Polarization in Ischemic Stroke by Targeting eEF1A1

**DOI:** 10.3389/fphar.2021.725268

**Published:** 2021-09-07

**Authors:** Yulan Liu, Shanshan Deng, Zhibing Song, Qian Zhang, Yuchen Guo, Yongsheng Yu, Yuliang Wang, Tiejun Li, Fayed A. K. Megahed, Tamer A. Addissouky, Junqin Mao, Yuefan Zhang

**Affiliations:** ^1^ School of Medicine, Shanghai University, Shanghai, China; ^2^ Department of Pharmacy, The Air Force Hospital From Eastern Theater of PLA, Nanjing, China; ^3^ College of Pharmacology, Anhui University of Chinese Medicine, Hefei, China; ^4^ Joint International Research Laboratory of Metabolic and Developmental Sciences, Key Laboratory of Urban Agriculture (South) Ministry of Agriculture, Plant Biotechnology Research Center, Fudan-SJTU-Nottingham Plant Biotechnology R&D Center, School of Agriculture and Biology, Shanghai Jiao Tong University, Shanghai, China; ^5^ Nucliec Acid Research Departement, Genetic Engineering and Biotechnological Research Institute, City of Scientific Research and Technological Applications, Alexandria, Egypt; ^6^ MLS Ministry of Health, Alexandria, Egypt; ^7^ Department of Clinical Pharmacy, Jiading Branch of Shanghai General Hospital, Shanghai Jiao Tong University School of Medicine, Shanghai, China

**Keywords:** brain ischemia, microglia polarization, inflammation, monocyte locomotion inhibitory factor (MLIF), eukaryotic translation elongation factor 1A1 (eEF1A1)

## Abstract

Monocyte locomotion inhibitory factor (MLIF) is a heat-stable pentapeptide from *Entamoeba histolytica*. Our previous study found that MLIF protects against ischemic stroke in rats and mice and exerts a neuroprotection effect in human neuroblastoma SH-SY5Y cells. Microglia/macrophage polarization has been proven to be vital in the pathology of ischemic stroke. Nevertheless, whether MLIF is able to modulate microglia/macrophage polarization remains unclear. We performed middle cerebral artery occlusion (MCAO) on C57BL/6J male mice and induced cultured BV2 microglia by oxygen-glucose deprivation (OGD), respectively. Immunfluorescence was utilized to detect the M1/2 markers, such as CD206 and CD16/32. qPCR and ELISA were used to detect the signature gene change of M1/2. The MAPK and NF-κB pathway associated proteins were measured by Western blot. To identify the protein target of MLIF, a pull-down assay was performed. We found that MLIF promoted microglia transferring from a “sick” M1 phenotype to a “healthy” M2 phenotype *in vivo* or *in vitro*. Furthermore, we proved that eukaryotic elongation factor 1A1 (eEF1A1) was involved in the modulation of microglia/macrophage polarization. Knocking down eEF1A1 by siRNA exhibited the M1 promotion effect and M2 inhibition effect. Taken together, our results demonstrated MLIF modulated microglia/macrophage polarization by targeting eEF1A1 in ischemic stroke.

## Introduction

Stroke is one of the three leading causes of death and also the most common reason for adults’ disability worldwide (Campbell and Khatri, 2020). Among all types of stroke, ischemic stroke, caused by blood vessel occlusion, accounts for the majority (Barthels and Das, 2020). Mechanisms involved in the process of stroke are extremely complicated, and inflammation is an important mediator in the pathogenesis of secondary injury after ischemic stroke (Jayaraj et al., 2019; Parikh et al., 2020).

As resident immune cells in the central nervous system, microglia are one of the first responders in CNS injuries (Greenhalgh et al., 2020; Xie et al., 2020). Currently, numerous studies have suggested that activated microglia with distinct phenotypes are double-edged swords in ischemic stroke (Liu ZJ. et al., 2019; Zhang et al., 2019; Lian et al., 2020). Differential polarization of microglia could likely explain the biphasic role of microglia in brain ischemia. Induced by IFN-γ and LPS stimulation, M1 phenotype microglia display enhanced expression of CD16/32, CD86, and iNOS and production of multiple pro-inflammatory cytokines (IL-1β, IL-6, and TNF-α) (Jha et al., 2016; Subhramanyam et al., 2019). The M2 phenotype could be induced by IL-4 or IL-13 and is characterized by enhanced expression of arginase-1, Fizz1, Ym1, CD206, insulin-like growth factor (IGF-1), and anti-inflammatory cytokines (IL-10, IL-4, and IL-13) (Prinz et al., 2019; Kwon and Koh, 2020). The M1 phenotype aggravates inflammation and exacerbates neuronal death, while the M2 phenotype contributes to improving neuronal survival and tissue repair (Han et al., 2021; Zhang et al., 2021). Thus, switching microglia from the M1 phenotype toward the M2 phenotype is an effective therapeutic strategy for ischemic stroke (Jiang et al., 2020).

Monocyte locomotion inhibitory factor (MLIF), a heat-stable pentapeptide produced by *Entamoeba histolytica* (Utrera-Barillas et al., 2003), had been approved as an investigational new drug for potential neural protection in acute ischemic stroke (Liu et al., 2018).

Our previous study found that MLIF could significantly decrease the cerebral infarction in the MCAO mice and rats and mitigate the ICAM-1 and VCAM-1 expression in OGD- or ox-LDL–induced bEnd3 cells by targeting eEF1A1 (Zhang et al., 2012). MLIF analogs have the same effect (Yao et al., 2011). Moreover, MLIF could also inhibit OGD-induced apoptosis in SH-SY5Y neuron cells by targeting eEF1A2 (Zhu et al., 2016). However, whether MLIF could regulate microglia polarization in ischemic stroke remains unclear.

In the current research, we explored a further study on the microglia/macrophage polarization of MLIF and found that MLIF promoted microglia polarization *in vivo* and *in vitro*. Moreover, this modulation effect is related to eEF1A1, the binding protein of MLIF in BV2 cells. It has been reported that eEF1A1 mediates the secretion of inflammatory cytokines in NF-κB signaling pathways, such as IL-1β, IL-18, and IL-6 (Wei et al., 2019). Therefore, MLIF targets eEF1A1 to regulate microglia polarization, probably through inflammatory pathways. Our current study revealed a novel pharmacological mechanism of MLIF that protects against ischemic stroke *via* modulating microglia polarization by targeting eEF1A1.

## Materials and Methods

### Reagents

MLIF and biotinylated MLIF were synthesized by Chinese Peptide Company (Hangzhou, China), with a purity above 98%. MLIF was dissolved in PBS (pH 7.4) to a final concentration of 4 mg/ml and stored at −80°C. eEF1A1 antibody was purchased from Abcam (Cambridge, MA, United States), and JNK, p-JNK, ERK, p-ERK, p38, p-p38, iκB, p-iκB, p65, and p-p65 antibodies were purchased from Cell Signaling Technology (Danvers, MA, United States). IRDye 800CW secondary antibody was purchased from LI-COR (Lincoln, NE, United States). Anti-Iba1 antibody was purchased from Wako (Tokyo, Japan). CD16/32 and CD206 antibodies were purchased from R&D Systems (MN, United States).Secondary antibodies (Cy™3 AffiniPure Donkey Anti-Rabbit IgG and Alexa Fluor488 AffiniPure Donkey Anti-Goat IgG) were purchased from Jackson ImmunoResearch Laboratories (West Grove, PA, United States).

### Middle Cerebral Artery Occlusion

C57BL/6J male mice weighting 25–30 g were purchased from Changzhou Cavens Experimental Animals Co., Ltd. (Changzhou, China). The mice were housed in a controlled environment under a 12-h light/dark cycle and received standard rat chow and water. All animal experiments and surgical procedures were supervised by the Institutional Animal Care and Use Committee at Shanghai University and conformed to international guidelines for the ethical use of experimental animals.

Male C57BL/6J mice were randomly divided into three groups (*N* = 10/group): the sham group, the MCAO group, and the MLIF treatment group. For the MLIF treatment group, MLIF (1 mg/kg) was injected into the tail vein 5 min prior to reperfusion. The other groups were treated with an equal volume of saline solution.

Transient MCAO surgery was preformed as previously described (Zhang et al., 2012). Briefly, mice were anesthetized with 2% sodium pentobarbital (3 ml/kg, i.p.) during the surgery. The left common carotid artery was placed into an 11-mm silicone-coated 8–0 filament from the internal carotid artery for 2 h, and the filament was pulled out gently to accomplish 24 h of reperfusion thereafter. Body temperature was maintained at 37.5 ± 0.5°C using a heating lamp. After reperfusion for 24 h, mice were euthanized, and the brains were harvested.

### Immunofluorescence

The brain slices were prepared as previously published (Zhang et al., 2016). After they were blocked with 5% goat serum in TBST, the brain slices were incubated with primary antibodies including anti-Iba1, anti-CD16/32, or anti-CD206 overnight at 4°C. After they were washed with PBS, the brain slices were incubated with Alexa Fluor488 donkey anti-mouse (1:800; Jackson ImmunoResearch Laboratories, West Grove, PA, United States) and Cy3-conjugated donkey anti-rabbit (1:800; Jackson ImmunoResearch Laboratories, West Grove, PA, United States) for 1 h at room temperature. DAPI solution (Thermo Fisher, Waltham, MA, United States) was used to stain nuclei. All images were processed using Image J (NIH Image, Bethesda, MD, United States) for counting of recognized cells. Three randomly selected microscopic fields were calculated in the peri-infarct cortex of each section. The data were expressed as the colocalization percent of CD16/32 and Iba1 or CD206 and Iba1.

### Cell Culture

BV2 microglia were obtained from the Cell Bank of the Chinese Academy of Sciences (Shanghai, China) and cultured in Dulbecco’s modified Eagle’s medium (DMEM) (Hyclone, Logan, UT, United States) with 10% fetal bovine serum (Gibco, Carlsbad, CA, United States) and 100 μg/ml penicillin–streptomycin solution (Thermo Scientific, Waltham, MA, United States). Cells were grown in 5% CO_2_ and 95% atmosphere at 37°C. The medium was replaced every 2 days.

### Oxygen-Glucose Deprivation

The OGD insult was carried out in BV2 cells to mimic ischemic conditions *in vitro* as described previously (Zhu et al., 2016). The culture medium was replaced with a glucose-free DMEM before OGD, cells were then incubated in a modular hypoxia chamber (Billups-Rothenberg, Del Mar, CA, United States) flushed with 5% CO_2_ and 95% N_2_. The chamber was placed in an incubator for 4 h at 37°C. The normal medium under normoxia served as the control. MLIF (1.0 μg/ml) was given prior to the OGD insult.

### Quantitative Reverse Transcription Polymerase Chain Reaction

Total RNA was extracted from brain tissues and BV2 cells using a Total RNA Kit (Takara, Shiga, Japan). cDNAs were synthesized using 5×Primescript reverse transcription reagents (Takara, Shiga, Japan) according to the manufacturer’s instruction. qRT-PCR was performed using SYBR Premix ExTaq (Tli RnaseH Plus) (Takara, Shiga, Japan) on a 7,500 Real-Time PCR System (Applied Biosystems). The levels of all target genes were normalized to the control (GAPDH). Primers used for quantitative RT-PCR (qRT-PCR) were as follows:

Mouse CD11b Forward: CCA​AGA​CGA​TCT​CAG​CAT​CA
Reverse: TTC​TGG​CTT​GCT​GAA​TCC​TT
Mouse CD32 Forward: AAT​CCT​GCC​GTT​CCT​ACT​GAT​C
Reverse: GTG​TCA​CCG​TGT​CTT​CCT​TGA​G
Mouse CD86 Forward: GAC​CGT​TGT​GTG​TGT​TCT​GG
Reverse: GAT​GAG​CAG​CAT​CAC​AAG​GA
Mouse iNOS Forward: GGC​AGC​CTG​TGA​GAC​CTT​TG
Reverse: GCA​TTG​GAA​GTG​AAG​CGT​TTC
Mouse CD206 Forward: TTC​GGT​GGA​CTG​TGG​ACG​AGC​A
Reverse: ATA​AGC​CAC​CTG​CCA​CTC​CGG​T
Mouse Arg-1 Forward: GAA​CAC​GGC​AGT​GGC​TTT​AAC
Reverse: TGC​TTA​GCT​CTG​TCT​GCT​TTG​C
Mouse Ym1 Forward: CGA​GGT​AAT​GAG​TGG​GTT​GG
Reverse: CAC​GGC​ACC​TCC​TAA​ATT​GT
Mouse Fizz1 Forward: CTG​CTA​CTG​GGT​GTG​CTT​GT
Reverse: GCA​GTG​GTC​CAG​TCA​ACG​AG
Mouse GAPDH Forward: CTT​CAC​CAC​CAT​GGA​GAA​GGC
Reverse: GGC​ATG​GAC​TGT​GGT​CAT​GAG

### Enzyme-Linked Immunosorbent Assay

ELISA was used to determine the inflammatory cytokines (IL-1β, TNF-α, IL-4, and IL-10) in the ischemic brain tissue homogenates and supernatant of the BV2 cells according to the protocol of the manufacturer’s kit (R&D Systems, MN, United States). The reactions were performed in ELISA plates and were read at a wavelength of 450 nm. Concentrations of inflammatory cytokines were obtained from the standard curve.

### Western Blot Analysis

BV2 cells were lysed with M-PER Protein Extraction Reagent (Pierce, Rockford, IL) supplemented with a protease inhibitor cocktail. The total protein concentration in the lysates was measured using BCA assay (Pierce, Rockford, IL). An equal amount of the extracts was analyzed in SDS-PAGE and subsequently electro-transferred onto PVDF membranes (Millipore, Temecula, CA, United States). After blocking with 5% BSA for 2 h, the membranes were incubated with the primary antibodies overnight at 4°C. The blots were washed and incubated with appropriate IRDye 800CW secondary antibodies (LI-COR, Lincoln, Nebraska, United States) for 1 h at room temperature. After being washed, the blots were scanned using an Odyssey scanner (LI-COR, Lincoln, Nebraska, United States). GAPDH was used as a loading control. The optical densities of the protein bands were semi-quantified using Quantity One software.

### Immunoprecipitation

Immunoprecipitation was performed as previously described (Zhang et al., 2012). BV2 cells were lysed with M-PER Protein Extraction Reagent (Pierce, Rockford, IL) supplemented with a protease inhibitor cocktail. The protein supernatant was incubated with the biotinylated MLIF (40 μl, 5 mg/ml) or the control solution for 6 h. After being washed twice with 1 ml of PBS, the streptavidin–agarose beads (Invitrogen, Carlsbad, CA, United States) were added and stirred gently at 4°C for 8 h. After it was washed twice with 1 ml of PBS, the mixture was centrifugated at 12,000 g for 5 min, and the first supernatant sample was obtained (S1). Then the supernatant was repeated using the binding assay twice to obtain sample 2 (S2) and sample 3 (S3). The control groups were correspondingly named C1, C2, and C3. After protein denaturation, the samples were separated by SDS-PAGE, followed by Coomassie brilliant blue staining.

### RNA Interference

eEF1A1 siRNA and control siRNA were obtained from Santa Cruz Biotechnology (Santa Cruz, CA, United States). The BV2 cells were transfected with eEF1A1 siRNA or control siRNA (50 nM) using INTERFERin^®^ Transfection Reagent (Polyplus, Illkirch, France) according to the manufacturer’s instructions. After 48 h of transfection, cells were lysed. Knockdown effect of eEF1A1 was analyzed by Western blot.

### Statistical Analysis

All data were expressed as the mean ± SD. Statistical analysis was performed using one-way analysis of variance (ANOVA). *p* < 0.05 was considered as statistically significant.

## Results

### Monocyte Locomotion Inhibitory Factor Promoted Microglia Polarization Toward the M2 Phenotype in the Middle Cerebral Artery Occlusion Model

**FIGURE 1:**
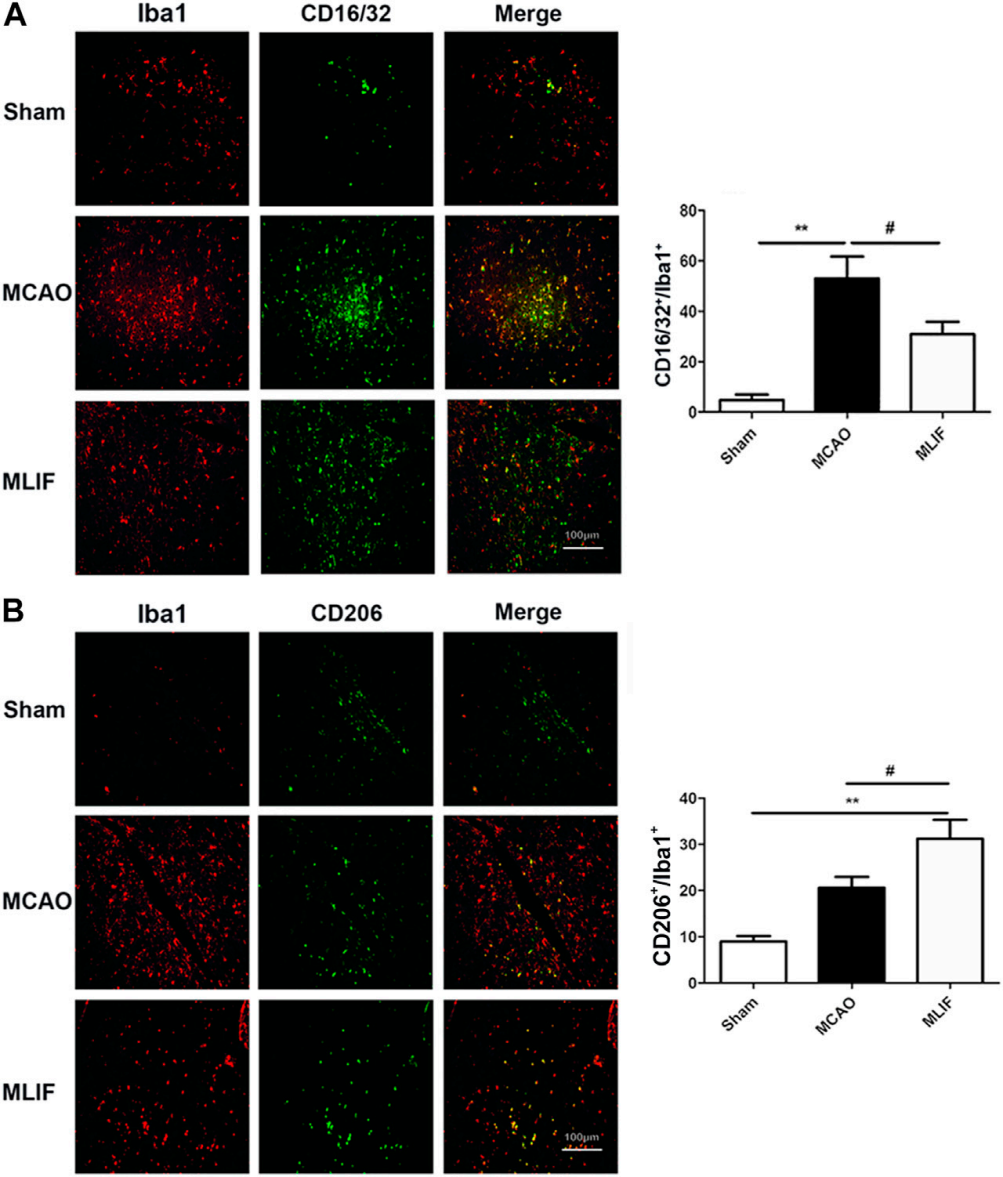
MLIF promoted microglia polarization toward the M2 phenotype in the mice MCAO model. **(A,B)** Double immunostaining was used to assess microglia (Iba1^+^) and an M1-associated marker (CD16/32^+^) or an M2-associated marker (CD206^+^) in mouse brain slices. The summarized bar graphs showed the colocalization rate of CD16/32 or CD206 with the Iba1 number in the ischemic region in the cortex. Scale bar: 200 μm. Data are expressed as the mean ± SEM. *N* = 6. **p < 0.01, vs Sham group, ^#^ *p* < 0.05 vs MCAO group.

To evaluate the effect of MLIF on microglia polarization in ischemic stroke, co-immunostaining was performed with the representative M1 marker CD16/32 or M2 marker CD206 and the microglia/macrophage marker Iba1. As shown in Figure 1A, the percentage of the CD16/32^+^ Iba1^+^ cells was higher in MCAO mouse brain slices (*p < 0.01*). The colocalization was obviously less in the MLIF group (*p < 0.05*). In contrast, the CD206^+^ Iba1^+^ cells were more in the MLIF group than the MCAO group (*p < 0.05,* Figure 1B). Together, these results suggested that MLIF promoted microglia polarization to the M2 phenotype after MCAO.

### Monocyte Locomotion Inhibitory Factor Promoted Microglia Polarization Toward the M2 Phenotype in Oxygen-Glucose Deprivation–Insulted BV2 Microglia

**FIGURE 2:**
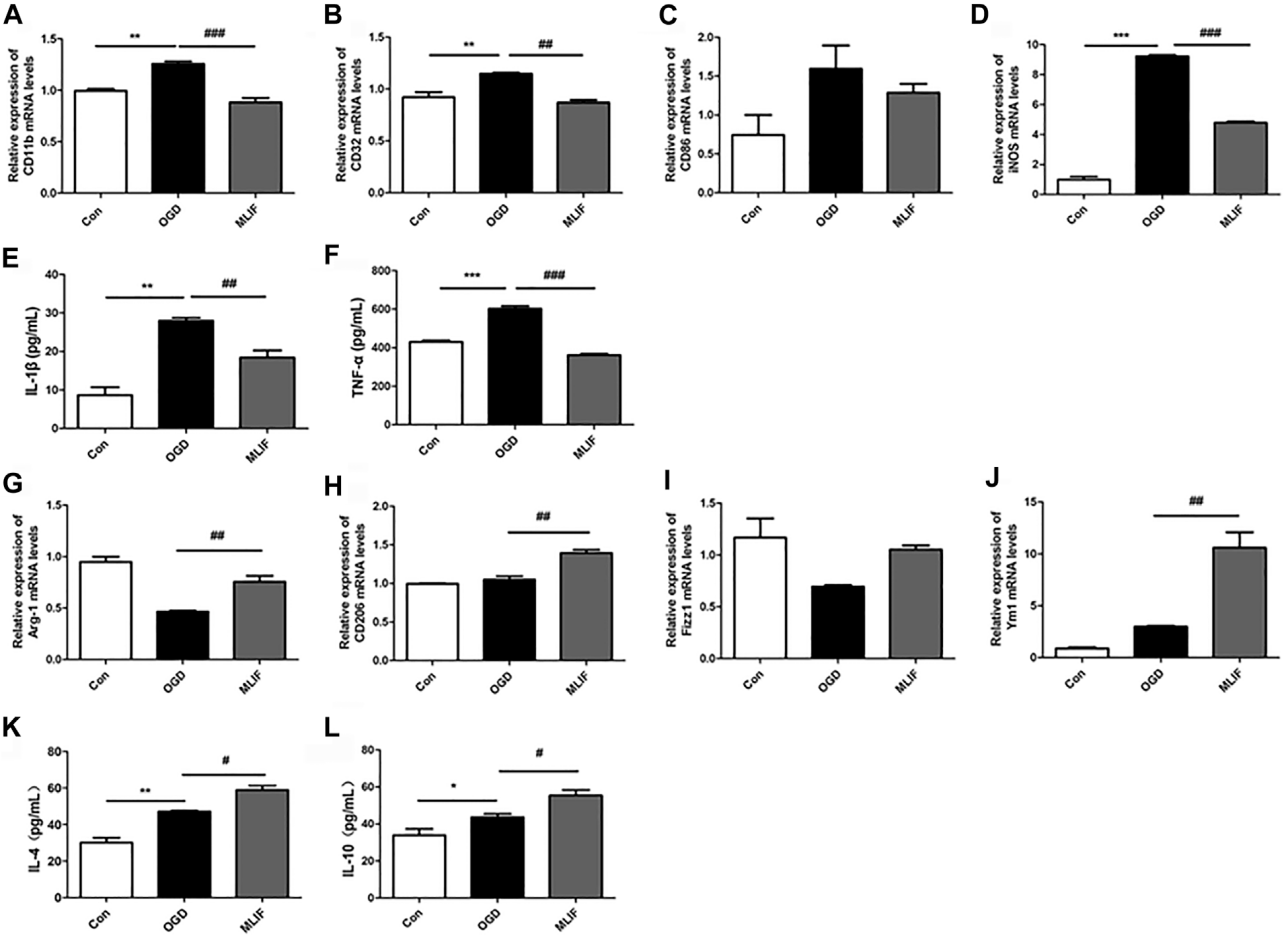
MLIF promoted microglia polarization toward M2 polarization in OGD-insulted BV2 microglia. BV2 cells were exposed to OGD for 4 h with or without MLIF (1.0 μg/ml) incubation. Real-time PCR was used to detect the M1 signature genes CD11b **(A)**, CD32 **(B)**, CD86 **(C)**, and iNOS **(D)** and the M2 signature genes Arg-1 **(G)**, CD206 **(H)**, Ym1 **(I)**, and Fizz1 **(J)**. ELISA was used to detect M1-related inflammation cytokines IL-1β **(E)** and TNF-α **(F)** and M2-related cytokines IL-4 **(K)** and IL-10 **(L)** in the cell supernatant. Data are expressed as the mean ± SEM. *N* = 3. *p < 0.05, **p < 0.01, ***p < 0.001, vs Control group; ^#^ *p* < 0.05, ^##^ *p* < 0.01, ^###^ *p* < 0.001, vs OGD group.

To further confirm the effect of MLIF on microglia polarization, we cultured BV2 microglia and established the OGD model to mimic ischemic conditions *in vitro*. Real-time PCR and ELISA were used to evaluate the microglia polarization effect of MLIF. As shown in Figures 2A–D, M1 signature genes including CD11b, CD32, and iNOS significantly increased compared with those in the control group (*p < 0.01, p < 0.001*), and MLIF significantly decreased the mRNA levels of CD11b, CD32, and iNOS (*p < 0.01, p < 0.001*). Meanwhile, MLIF also reduced production of inflammatory cytokines such as IL-1β and TNF-α (*p < 0.01, p < 0.001,* Figures 2E,F). Moreover, the MLIF-treated group exhibited higher mRNA expression of M2 signature genes including Arg-1, CD206, Ym1, and Fizz1 and more M2-related cytokines including IL-4 and IL-10 than the OGD group (Figures 2G–L). Taken together, *in vitro* data demonstrated that MLIF promoted M2 polarization and inhibited M1 polarization in OGD-insulted BV2 microglia.

## Monocyte Locomotion Inhibitory Factor Inhibited Oxygen-Glucose Deprivation–Insulted BV2 Microglia M1 Polarization by Suppressing the NF-κB Pathway

**FIGURE 3:**
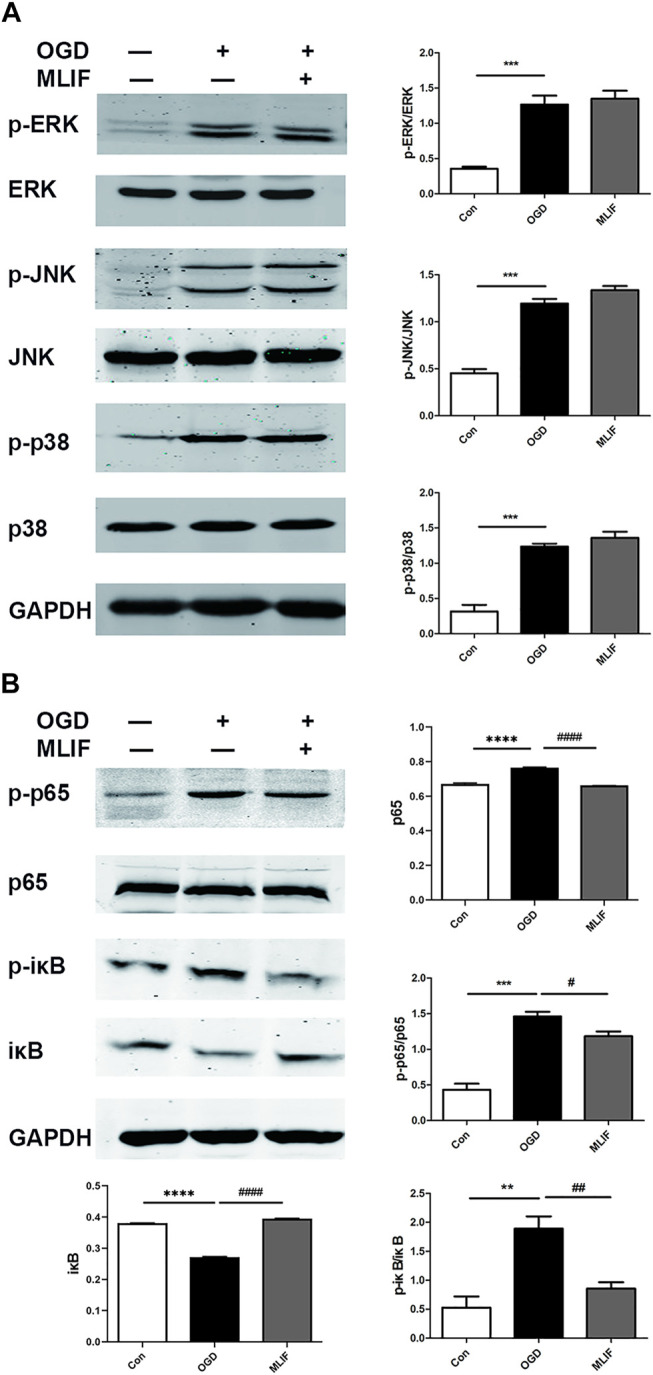
Effect of MLIF on the MAPK pathway and the NF-κB pathway in OGD-insulted BV2 microglia. BV2 cells were exposed to OGD for 4 h with or without MLIF (1.0 μg/ml) incubation. **(A)** MAPK pathway–associated proteins p-ERK, p-JNK, and p-p38 and **(B)** NF-κB pathway–associated proteins p65, p-p65, iκB, and p-iκB were determined by Western blot. Data are expressed as the mean ± SEM. Results were analyzed using one-way ANOVA; *N* = 3. **p < 0.01, ***p < 0.001, ****p < 0.0001, vs Control group; ^#^ *p* < 0.05, ^##^ *p* < 0.01, ^####^ *p* < 0.0001, vs OGD group.

It was reported that MLIF regulates 385 genes which are involved in the MAPK and NF-κB pathways (Silva-García et al., 2008). So we evaluated the effect of MLIF on related proteins of the MAPK and NF-κB pathways in OGD-insulted BV2 microglia. Western blot results showed that MLIF had no significant effect on the MAPK pathway (Figure 3A). However, the p-p65 and p-iκB expression was significantly increased in the OGD group (*p < 0.01, p < 0.001*), whereas MLIF significantly inhibited their expression (*p < 0.05, p < 0.01*, Figure 3B). Thus, MLIF could suppress the OGD-induced NF-κB pathway activation in OGD-insulted BV2 microglia.

### Monocyte Locomotion Inhibitory Factor Binds to Eukaryotic Translation Elongation Factor 1A1 in BV2 Microglia

**FIGURE 4:**
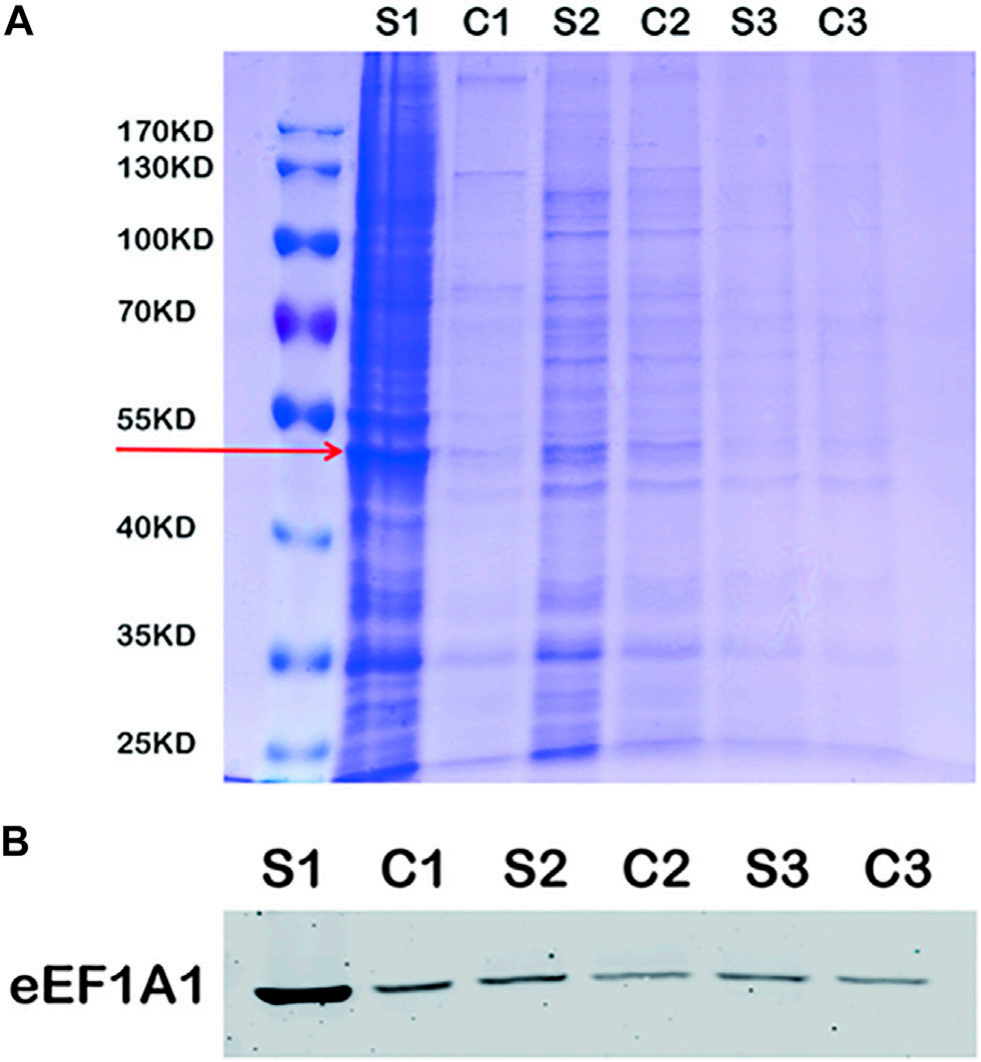
eEF1A1 is the binding protein of MLIF in BV2 microglia. **(A)** After immunoprecipitation, the binding proteins were separated by SDS-PAGE and stained with Coomassie brilliant blue. **(B)** Specific binding protein was confirmed by Western blotting in BV2 microglia.

The immunoprecipitation was used to find the binding protein of MLIF in BV2 microglia. As shown in Figure 4A, the specific binding protein (red arrow) appeared at ∼50 kDa in bio-MLIF groups (S1, S2, and S3) compared with control groups (C1, C2, and C3). Based on our previous study (Zhang et al., 2012), we speculated that it was eEF1A1. Then, Western blot analysis was performed with the specific anti-eEF1A1 antibody, and it was finally confirmed that the 50-kDa protein was eEF1A1 (Figure 4B).

### Eukaryotic Translation Elongation Factor 1A1 Is Responsible for the Microglia Polarization Effect of Monocyte Locomotion Inhibitory Factor

**FIGURE 5:**
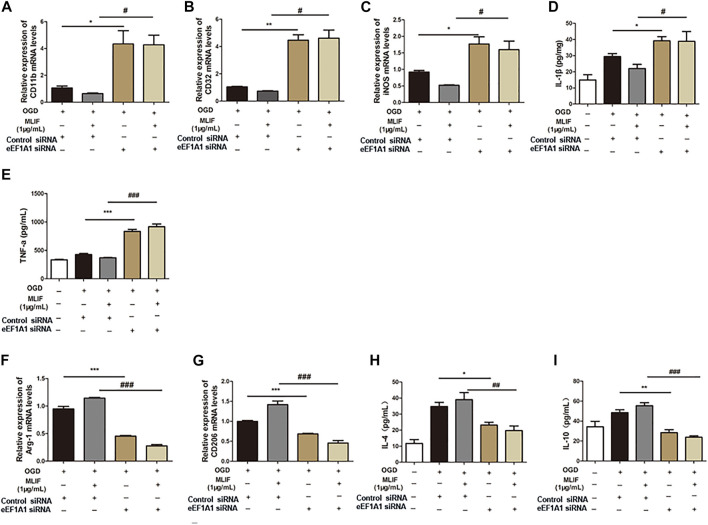
Regulation effect provided by MLIF was inhibited by eEF1A1 siRNA in BV2 microglia. OGD-insulted BV2 microglia transfected with eEF1A1 siRNA or control siRNA were treated with MLIF (1.0 μg/ml). Real-time PCR was used to detect M1 signature genes CD11b **(A)**, CD32 **(B)**, and iNOS **(C)** and M2 signature genes Arg-1 **(F)** and CD206 **(G)**. ELISA was used to detect M1-related inflammation cytokines IL-1β **(D)** and TNF-α **(E)** and M2-related cytokines IL-4 **(H)** and IL-10 **(I)** in the cell supernatant. Data are expressed as the mean ± SEM. *N* = 3. *p < 0.05, **p < 0.01, ***p < 0.001, vs OGD + Control siRNA group; ^#^ *p* < 0.05, ^###^ *p* < 0.001, vs MLIF + Control siRNA group.

**FIGURE 6:**
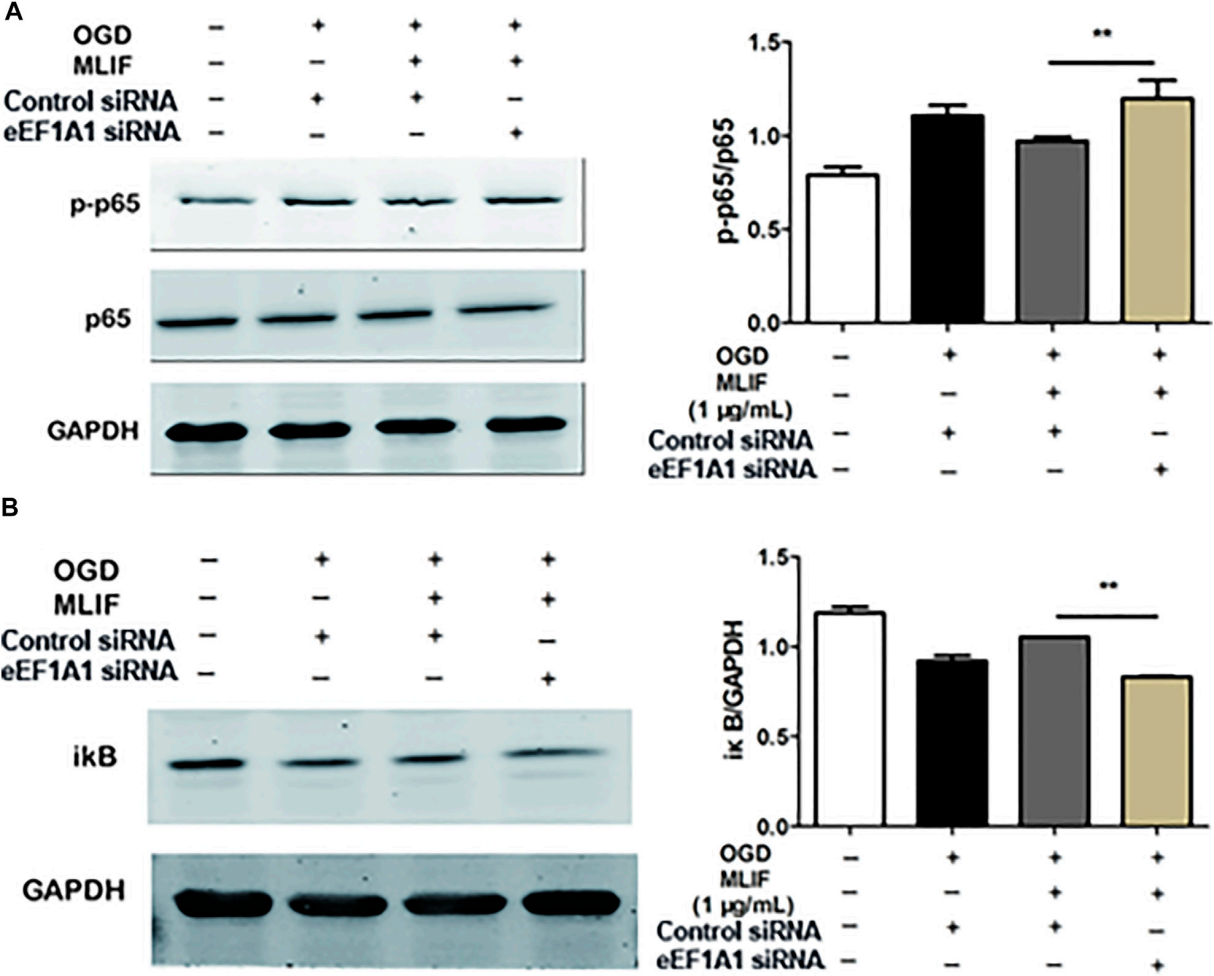
MLIF suppressed the NF-κB pathway *via* eEF1A1. After knock-down of eEF1A1 by using eEF1A1 siRNA or control siRNA, OGD-exposed BV2 microglia were incubated with MLIF (1.0 μg/ml). The levels of p-p65 **(A)** and iκB **(B)** were evaluated by immunoblotting. Data are expressed as the mean ± SEM. *N* = 3. **p < 0.01 vs Control siRNA group.

To further examine the microglia polarization effect of MLIF, we used siRNA to knock down the expression of eEF1A1 in BV2 microglia. Real-time PCR and ELISA were used to determine the M1 or M2 signature genes and related cytokines. As expected, knock-down of eEF1A1 attenuated the M1 inhibitory effect (Figure 5A-E) and the M2 promotive effect of MLIF (Figures 5F–I). As shown in Figure 6, the inhibitory effect of MLIF on p-p65 and the promotive effect of MLIF on iκB were blocked by eEF1A1 siRNA. Therefore, we drew the conclusion that eEF1A1 was involved in the microglia polarization modulation of MLIF.

## Discussion

MLIF has been proven to have protection in rat and mice ischemic stroke models in our previous study (Zhang et al., 2012). Our recent study has demonstrated that MLIF exhibits neuroprotection in OGD-insulted SH-SY5Y neuroblastoma by targeting eEF1A2 (Zhu et al., 2016). But the mechanisms underlying the anti-inflammation effects of MLIF have not been fully explored yet. As we all know, microglia are the first cells to respond to CNS injuries and are potent modulators of CNS repair and regeneration. Currently, a great deal of the literature suggests that microglia with distinct phenotypes are apparently double-edged swords in the battle for brain ischemia (Liu ZJ. et al., 2019; Zhang et al., 2019). Usually, M1 phenotype microglia accelerate the death of neurons and aggravate inflammation, while the M2 phenotype contributes to improving neuronal survival and tissue repair (Han et al., 2021). With the properties of anti-inflammation, we speculated that MLIF may protect ischemic stroke by regulating the microglia polarization.

Hu et al. (2015) reported that microglia/macrophages respond dynamically to ischemic injury, experiencing an M2 phenotype, followed by a transition to an M1 phenotype. In the current study, our immunofluorescence results have demonstrated that MLIF inhibited the expression of CD16/32^+^Iba1^+^ and promoted the expression of CD206^+^Iba1^+^ in the MCAO mice brain. We also confirm the modulation effect of MLIF in OGD-insulted BV2 microglia *in vitro*, and we also found that MLIF promoted the M2 phenotype and inhibited the M1 polarization of BV2 microglia, which was demonstrated by changes in the expression of M1/2 signature genes and related cytokines.

It has been reported that MLIF could regulate multitudinous gene expression in U937 cells, which were involved in the MAPK and NF-κB pathways (Silva-García et al., 2008). The mitogen-activated protein kinases (MAPKs), including three major subfamilies, the extracellular signal-regulated kinase (ERK), c-Jun N-terminal kinase (JNK) pathway, and p38, are a family of serine/threonine protein kinases that mediate fundamental biological processes and cellular responses to external stress signals (Kassouf and Sumara, 2020). The MAPK pathway participates in M1 macrophage polarization (He et al., 2020). Nuclear factor-κB (NF-κB) is a transcription factor that regulates the expression of genes that control cell proliferation and apoptosis, as well as genes that respond to inflammation and immune responses (Jimi et al., 2019). Inhibition of NF-κB could promote macrophages toward M2 polarization (Ye et al., 2019). Previous study has suggested that the process of macrophage M2 activation is mediated by the NF-κB and STAT3 pathways (Qian et al., 2020). Our results indicate that MLIF could suppress M1 microglia polarization by inhibiting NF-κB pathway activation in OGD-insulted BV2 microglia but has no significant effect on the MAPK pathway. To further investigate the mechanism of MLIF in regulating microglia polarization, we subsequently identify that the MLIF targeting protein was eEF1A1 by immunoprecipitation. Thus, it is presumable that eEF1A1 is involved in microglia polarization.

Eukaryotic translation elongation factor 1 alpha (eEF1A), a member of the G protein family, could interact with aminoacyl tRNA (aa-tRNA) and transfer it to the acceptor site of the ribosome during the elongation cycle in peptide synthesis (Knight et al., 2020). eEF1A has two protein isoforms in mammals, encoded by separate genes, which are 92% identical and 98% similar at the amino acid level (McLachlan et al., 2019). However, eEF1A2 is expressed in a tissue-specific manner, and eEF1A is expressed ubiquitously (Li et al., 2019). In addition to its roles in polypeptide chain elongation, the noncanonical functions of eEF1A1 have become a focus recently, such as apoptosis (Akintade and Chaudhuri, 2020), virus infection (White et al., 2021), signal transduction (Akintade and Chaudhuri, 2020), and tumorigenesis (Liu S. et al., 2019). Our previous study indicated that MLIF decreased the ICAM-1 and VCAM-1 expression and increased the eNOS expression in OGD- or ox-LDL–induced bEnd3 cells by targeting eEF1A1 (Zhang et al., 2012). It was reported that inactivation of eEF1A proteins leads to immunodeficiency and neural and muscular defects and favors apoptosis (Abbas et al., 2015). Recently, reports have suggested that the eEF1A1 protein may have therapeutic relevance in diverse conditions with altered neurite outgrowth (McLachlan et al., 2019; Duman et al., 2020). A recent study has suggested that eEF1A1 and CagA cooperated to mediate the expression of IL-6 by affecting the activity of p-STATS727 in the nucleus (Xu et al., 2020). Our previous study reported that eEF1A1/HSC70 cooperatively suppress brain endothelial cell apoptosis *via* regulating JNK activity (Liu et al., 2016). These studies suggested that eEF1A1 has a crucial role in neuron protection and regulation of inflammatory cytokines and adhesion molecules. Thus, we speculated that eEF1A1 may exert an important effect in regulating microglia polarization in ischemic stroke.

To confirm the importance of eEF1A1 in mediating effects of MLIF, we knocked down eEF1A1 in BV2 microglia by utilizing its siRNA. The results suggested that inhibition of eEF1A1 results in the M1 promotion effect and the M2 suppression effect on OGD-insulted BV2 microglia. What is more, the inhibition effect of MLIF on the NF-κB pathway was also attenuated. Our results suggested that the eEF1A1/NF-κB pathway plays a critical role in the microglia polarization regulated by MLIF. It also revealed the novel pharmacological effect of eEF1A1, participating in the microglia polarization in ischemic stroke.

In conclusion, the present results demonstrated that MLIF promoted microglia transition toward the M2 phenotype in the *in vivo* and *in vitro* ischemic stroke model. An NF-κB signaling pathway was involved in the pharmacological action of MLIF by targeting eEF1A1.

## Data Availability

The raw data supporting the conclusions of this article will be made available by the authors, without undue reservation.
